# Machine Learning Driven Discovery of Ribosomal Biomarkers in PCOS

**DOI:** 10.1093/bib/bbag056

**Published:** 2026-03-10

**Authors:** Ashitha Washington, Ravindra Kumar

**Affiliations:** Computational Biology and Bioinformatics Lab, Department of Bioscience and Engineering, National Institute of Technology Calicut, Kozhikode, Kerala 673601, India; Computational Biology and Bioinformatics Lab, Department of Bioscience and Engineering, National Institute of Technology Calicut, Kozhikode, Kerala 673601, India

## Abstract

Polycystic ovary syndrome (PCOS) represents a multifaceted endocrine condition marked by genetic, molecular, and phenotypic variability. To uncover consistent transcriptomic biomarkers and prognostic gene networks linked to PCOS, we performed an integrative analysis of RNA-Seq data compiled from publicly available Gene Expression Omnibus datasets, comprising 65 PCOS cases and 61 healthy controls across diverse cell types. Data preprocessing involved normalization followed by differential expression analysis. Feature selection was then performed via Elastic Net regression, effectively managing multicollinearity and refining the feature set to 83 candidate genes for subsequent modeling.

Multiple machine learning classifiers were trained and validated using a 60:20:20 data split, with hyperparameter optimization to enhance predictive performance. Among these, the Support Vector Machine (SVM) model exhibited the highest classification capability, achieving 92.31% accuracy on the internal validation set and an impressive AUC of 0.98. Model explainability was strengthened using SHAP and LIME analyses, pinpointing the most influential genes driving model predictions. Logistic regression based on the key gene clusters produced a prognostic framework with an AUC of 0.82 and precision of 0.8, suggesting their robustness as biomarkers despite PCOS heterogeneity.

Functional enrichment results revealed that these genes are predominantly involved in RNA-binding processes, ribosomal machinery, and immune regulation. Overall, this integrative multi-cohort analysis coupled with advanced machine learning provides a powerful strategy for identifying clinically actionable biomarkers and prognostic signatures in PCOS, offering new avenues for molecular diagnosis and therapeutic development.

**References**

1. Jiang B. “The Global Burden of Polycystic Ovary Syndrome in Women of Reproductive Age: Findings from the GBD 2019 Study”. IJWH 2025; Volume 17:153–165

2. Schwämmle V, Jensen ON. “A simple and fast method to determine the parameters for fuzzy c–means cluster analysis”. Bioinformatics 2010; 26:2841–2848

3. Zheng Y, Bian Y, Wu R, et al. “High-Throughput Sequencing Profiles About lncRNAs and mRNAs of Ovarian Granulosa Cells in Polycystic Ovary Syndrome”. Front. Med. 2021; 8:741803

